# Protective immunity against Chagas disease induced by a superantigen-based chimeric DNA vaccine delivered by attenuated *Salmonella*

**DOI:** 10.3389/fimmu.2026.1788924

**Published:** 2026-03-12

**Authors:** María Belén Antonoglou, Andrés Sánchez Alberti, Daniela María Redolfi, Augusto Ernesto Bivona, Sofía Noli Truant, María Belén Sarratea, Alejandro Cardoso, Flavia Nader Motta, Emilio Luis Malchiodi, Marisa Mariel Fernández

**Affiliations:** 1 Universidad de Buenos Aires, Facultad de Farmacia y Bioquímica, Departamento de Microbiología, Inmunología, Biotecnología y Genética, Cátedra de Inmunología, Buenos Aires, Argentina; 2 Instituto de Estudios de la Inmunidad Humoral "Prof. Ricardo A. Margni" (IDEHU), UBA-CONICET, Buenos Aires, Argentina; 3 Universidad de Buenos Aires, Facultad de Medicina, Departamento de Microbiología, Parasitología e Inmunología, and Instituto de Microbiología y Parasitología Médica (IMPaM), UBA-CONICET, Buenos Aires, Argentina

**Keywords:** Chagas disease, DNA vaccine, heterologous chimeric immunogen, polyfunctional T cells, protective Th1 immunity

## Abstract

**Introduction:**

Chagas disease is a chronic parasitic infection endemic to Latin America that affects more than 7 million people and is increasingly spreading worldwide due to human migration. *Trypanosoma cruzi* is the etiological agent of this disease, for which no effective vaccine has yet been approved for human use. We previously developed a heterologous chimeric immunogen, CruSEG, composed of the N-terminal domain of the major cysteine protease cruzipain (Nt-Cz) fused to a genetically detoxified mutant of the staphylococcal superantigen G (SEGN24A). This modified superantigen preserves innate immune activation while avoiding deleterious T-cell effects associated with native superantigens.

**Methods:**

Here, we evaluated the immunogenicity and protective efficacy of CruSEG using different vaccination strategies, including recombinant protein formulated with CpG-ODN,oral DNA immunization delivered by attenuated *Salmonella enterica*, and a heterologous prime–boost regimen combining both platforms.

**Results:**

All vaccination protocols induced Nt-Cz–specific immune responses, albeit with distinct qualitative profiles. Recombinant protein vaccination elicited robust humoral immunity characterized by Th1-skewed IgG2a responses and strong parasite neutralizing activity, whereas DNA delivery preferentially promoted potent cellular immunity, including polyfunctional CD4^+^ T cells and IFN-γ–producing CD8^+^ T cells. The prime–boost strategy generated a broader immune profile but did not confer superior protection compared with homologous regimens. Upon challenge with *T. cruzi* strains belonging to different discrete typing units, vaccinated mice exhibited significant reductions in parasitemia during the acute phase and sustained control of parasite burden and tissue damage during chronic infection.

**Discussion-conclusions:**

Although sterilizing immunity was not achieved, the attenuation of parasite persistence and pathology represents a biologically relevant outcome. Collectively, these findings demonstrate that incorporating a detoxified bacterial superantigen into chimeric antigens across multiple platforms enhances immune quality and protective efficacy, highlighting engineered superantigens as promising immune modulators for the development of effective vaccines against Chagas disease

## Introduction

Chagas disease, also known as American trypanosomiasis, is caused by the protozoan parasite *Trypanosoma cruzi*, which is primarily transmitted by blood-sucking triatomine insects. Historically restricted to endemic regions extending from the southern United States to central Argentina, Chagas disease has become a global public health concern as a consequence of human migration. In addition to vectorial transmission, *T. cruzi* infection can occur through blood transfusion and organ transplantation, vertical transmission from mother to child, and the ingestion of food or beverages contaminated with the parasite.

Clinically, Chagas disease comprises an acute and a chronic phase. The acute phase is often asymptomatic or presents with mild, nonspecific symptoms, contributing to its characterization as a “silent” infection. Individuals not treated during this phase usually progress to chronic infection, of whom approximately 30–40% develop clinically significant disease. Chagas cardiomyopathy is the most frequent and severe manifestation, affecting 14–45% of chronically infected patients (1), whereas gastrointestinal involvement is less common (10–21%) and more prevalent in the Southern Cone of South America (2).

Currently, only two drugs—benznidazole and nifurtimox—are licensed for the etiological treatment of Chagas disease. Although highly effective during the acute phase, their efficacy declines with increasing time since infection and they require prolonged regimens frequently associated with adverse effects, leading to high treatment discontinuation rates (2). As a result, antiparasitic therapy provides limited benefit for many chronically infected patients, underscoring the urgent need for alternative control strategies. In this context, the absence of an effective vaccine represents a major unmet medical need for the prevention and long-term control of *T. cruzi* infection, and no vaccine has yet been evaluated in human clinical trials.

Vaccination strategies using bacterial or viral vectors as antigen delivery systems have gained increasing relevance for Chagas disease [reviewed in 3, 4). These platforms provide intrinsic adjuvant activity through pathogen-associated molecular patterns (PAMPs) that activate antigen-presenting cells via pattern recognition receptors, thereby enhancing innate and adaptive immune responses (5, 6). In addition, attenuated microbial vectors are commonly administered via mucosal routes, inducing both mucosal and systemic immunity (7, 8). Among these, the attenuated *Salmonella enterica* serovar *Typhimurium aroA* strain SL7207 has been extensively validated as a DNA delivery vector in experimental models of infectious diseases, including *T. cruzi*, as well as in cancer immunotherapy (9–19).

Bacterial superantigens (SAgs) are a family of protein toxins with exceptionally potent immunostimulatory properties, characterized by their ability to simultaneously bind major histocompatibility complex class II molecules on antigen-presenting cells and T cell receptors on T lymphocytes. Due to their capacity to strongly modulate immune responses, superantigens have been explored as therapeutic tools in cancer and as immunomodulators to enhance vaccine efficacy (20).

Previously, we developed a heterologous chimeric immunogen combining a genetically detoxified bacterial superantigen (SEGN24A) (21, 22) with the catalytic N-terminal domain of the *T. cruzi* cysteine protease cruzipain (Nt-Cz) (23), designed to couple innate immune modulation with antigen-specific adaptive immunity. We demonstrated that the recombinant Cru-SEGN24A protein, adjuvanted with CpG-ODN, conferred significant protection against *T. cruzi* infection, outperforming the non-conjugated components (24). However, whether this chimeric immunogen could maintain its immunogenicity and protective efficacy when delivered as a DNA vaccine using a live bacterial vector remained unexplored. In the present study, we therefore evaluated the immunogenicity and protective efficacy of Cru-SEGN24A encoded in a eukaryotic expression plasmid pcDNA3.1, and delivered by attenuated *Salmonella enterica*, assessing humoral and cellular immune responses as well as protection against experimental *T. cruzi* challenge.

## Materials and methods

### Animals and parasites

In this study, inbred C3H/HeN and outbred CF-1 mice were used. Animals were bred and maintained at the animal facility of the Institute of Medical Microbiology and Parasitology (IMPaM, UBA–CONICET), Faculty of Medicine, University of Buenos Aires. All procedures involving animals were conducted in accordance with the ethical standards of the University of Buenos Aires and were approved by the Institutional Committee for the Care and Use of Laboratory Animals (CICUAL), Faculty of Pharmacy and Biochemistry, University of Buenos Aires (Resolution No. 3381-18). Animal sample size was estimated using a power-based method (25). Euthanasia has been performed by exsanguination in animals previously anesthetized with ketamine/xylazine, using a dose of 100mg ketamine/kg body weight and 10 mg xylazine/kg body weight, for sample collection (exsanguination). This procedure has been carried out at the Immunology Department’s animal facility.

Bloodstream trypomastigotes of *Trypanosoma cruzi* RA strain, K98 clone (derived from the CA-I strain), and the Tulahuen strain expressing *Escherichia coli* β-galactosidase (Tul β-gal) (26) were obtained from the blood of infected CF-1 mice. Heparinized blood was centrifuged at 1,000 × g for 5 min, incubated for 1 h at 37 °C and the trypomastigotes were subsequently recovered from the supernatant.

### Bacteria, plasmids, and antigens

Electrocompetent bacteria from different *E. coli* strains and attenuated *Salmonella* enterica serovar Typhimurium strains were transformed with the corresponding plasmids. Electroporation was performed using a Micropulser system (Bio-Rad, CA, USA) with 0.2 cm Gene Pulser cuvettes (Bio-Rad). Positive clones were selected on agar plates containing ampicillin.

The heterologous chimeric *ncz-segn24a* gene was constructed by splicing by overlap extension PCR (SOE-PCR), as previously described (24). The recombinant NCz-SEGN24A protein (69 kDa) was expressed in *E. coli* BL21 (DE3) as inclusion bodies, purified under denaturing conditions by Ni²^+^-NTA affinity chromatography, refolded *in vitro* by dialysis, and further purified by size-exclusion chromatography using a Superdex 200 column (GE Healthcare). Protein purity was assessed by SDS-PAGE. Endotoxin contamination was evaluated using the Pierce LAL Chromogenic Endotoxin Quantitation Kit (Thermo Scientific) according to the manufacturer’s instructions.

To perform immunizations with DNA encoding CruSEG, the corresponding gene was cloned into the eukaryotic expression plasmid pcDNA3.1(+). *cruseg* was previously cloned in pGEM T-easy vector serving as a template to amplify the *cruseg* gene by PCR using the following primers:

PFpcDNA: 5′-GCGC**GGATCC**GCGATGGCGCCCGCGGCAGTGGATT-3′.

PRpcDNA: 5′-CGCGCG**GAATTC**TTAGTGAGTATTAAGAAATAC-3′.

Restriction sites for BamHI and EcoRI are shown in bold, and the Kozak sequence (GXXATG), required for efficient translation initiation in eukaryotic cells, is underlined.

The PCR product was purified, digested with the corresponding restriction enzymes, and ligated into pcDNA3.1(+), previously digested with the same enzymes. The resulting pcDNA-cruseg construct was used to transform competent *E. coli* DH5α cells, which were grown on LB agar plates containing ampicillin (100 μg/mL). Ampicillin-resistant colonies were randomly selected, plasmids were extracted as previously described, and the presence of the *cruseg* insert was confirmed by PCR using specific primers and by restriction enzyme digestion. The integrity of the *cruseg* sequence in the pcDNA3.1(+) plasmid was further verified by DNA sequencing.

Recombinant plasmid pcDNA-cruseg was sequenced by Macrogen (Seoul, South Korea). Amino acid sequences were deduced by translating nucleotide sequences using the ExPASy Translate tool. Both nucleotide and amino acid sequences were compared with published *seg* and *nt-cz* gene sequences using BLAST (NCBI) and ClustalW for sequence alignment.

### Immunization protocols

For all experiments, groups of 5–6 male or female C3H/HeN mice (6–8 weeks old) were immunized with four doses administered at 10-day intervals. For DNA delivery using the *Salmonella* system, mice received oral immunizations every 10 days as follows: Group I (control) received 1 × 10^9^ CFU of *Salmonella enterica* serovar Typhimurium aroA SL7207 transformed with empty pcDNA3.1; Group II (SCruSEG) received the same dose of SL7207 carrying pcDNA-cruseg; Group III (CruSEG+CpG) was immunized intramuscularly with 10 μg of CruSEG protein co-adjuvanted with 10 μg CpG; Group IV (prime–boost) received two oral doses of SCruSEG followed by two intramuscular boosts with CruSEG plus CpG.

### ELISA for antigen-specific IgG determination

Sera were collected 15 days after the last immunization and antigen-specific IgG, IgG1, and IgG2a antibodies were quantified by indirect ELISA as previously described (14). Plates were coated with recombinant Nt-Cz protein and incubated with serially diluted sera. Bound antibodies were detected using HRP-conjugated anti-mouse IgG (Jackson ImmunoResearch) or biotinylated anti-mouse IgG1 or IgG2a antibodies (Pharmingen, BD). The reaction was developed using TMB as chromogen and the absorbance was measured at 450 nm.

### Cell invasion inhibition assay

Bloodstream trypomastigotes of *T. cruzi* Tulahuen strain expressing *E. coli* β-galactosidase (Tul β-gal) (26) were pre-incubated with heat-inactivated immune sera (1:10 dilution) for 30 min at 37°C and then added to Vero cell monolayers at a 10:1 parasite-to-cell ratio. After 18 h, non-invading parasites were removed, cultures were maintained for 4 days, and β-galactosidase activity was quantified using CPRG substrate. Invasion was expressed as percent inhibition relative to parasites incubated without serum, as previously described (26).

### Detection of CruSEG-specific antibodies by surface plasmon resonance

Antibody binding to CruSEG was analyzed by surface plasmon resonance (SPR) using a Biacore T100 biosensor (27). CruSEG was captured via immobilized anti-SEG IgG on a carboxymethyl-dextran sensor chip CM5, and sera from immunized mice were injected at serial dilutions. Binding responses were recorded as resonance units (RU), and nonspecific interactions were controlled using reference surfaces without immobilized ligand.

### Delayed-type hypersensitivity assay

Fifteen days after the final immunization, mice were injected intradermally into the hind footpad with 10 μg of Nt-Cz. Footpad thickness was measured before and 48 h after antigen challenge, and DTH responses were expressed as the difference in thickness (mm).

### Splenocyte isolation and *in vitro* assays

Splenocytes were obtained 15 days after the last immunization by mechanical dissociation of spleens, followed by red blood cell lysis. Cells were resuspended in RPMI supplemented with 10% fetal bovine serum and counted for subsequent assays.

Antigen-specific proliferation was evaluated by culturing splenocytes with Nt-Cz (10 μg/mL) for 96 h, followed by [³H]-thymidine incorporation during the final 18 h. Results were expressed as a proliferation index relative to unstimulated controls.

Cytokine secretion (IFN-γ, IL-12, IL-10, and IL-17) was measured in culture supernatants after 48 h of stimulation with Nt-Cz using commercial capture ELISA kits according to the manufacturers’ instructions (BD Biosciences, immunoassay ELISA Reagents).

### Flow cytometry analysis of T cell cytokine production

For intracellular cytokine staining, splenocytes were stimulated with Nt-Cz and, for CD8^+^ T cells, with the Nt-Cz-derived H-2K^k^-restricted peptide KEEASSAVV, in the presence of anti-CD28. Brefeldin A was added to block cytokine secretion. Cells were stained for surface CD4 and CD8, fixed, permeabilized, and stained intracellularly for IFN-γ, TNF-α, and IL-2. Data was acquired on a BD FACSCanto II flow cytometer and analyzed using standard compensation controls.

### Evaluation of protection against *T. cruzi* infection

Two weeks after the final immunization, mice were challenged intraperitoneally with bloodstream trypomastigotes. For lethal infection, mice received 1 × 10³ or 1 × 10^4^ RA strain parasites, and parasitemia was monitored every 2–3 days by microscopic counting of blood samples. Body weight and survival were recorded daily. For sublethal infection, mice were challenged with 3 × 10^5^ K98 clone parasites, and parasitemia was assessed weekly during the acute phase. At 100 days post-infection (dpi), animals were euthanized and sera and muscle tissue were collected for chronic phase analysis.

### Tissue damage, histopathology, and parasite load

As markers of chronic-phase tissue injury, serum levels of muscle enzymes were evaluated at 100 dpi. Creatine kinase (CK) and its cardiac-specific isoenzyme (CK-MB) activities were quantified in sera from infected mice. Enzymatic activities were determined in sera by a kinetic spectrophotometric assay based on NADPH oxidation, measuring absorbance at 340 nm, according to the manufacturer’s instructions (Wiener Lab, Argentina). Results were expressed as international units per liter (U/L). Heart and skeletal muscle samples were fixed, paraffin-embedded, sectioned, and stained with hematoxylin–eosin for blinded histopathological analysis.

Parasite burden in cardiac and skeletal muscle tissues was quantified by real-time PCR targeting T. cruzi satellite DNA (TcSat), using mouse TNF-α as an internal control, as previously described (28).

### Statistical analysis

Statistical analyses were performed using one- or two-way ANOVA with appropriate *post hoc* tests, or Student’s *t*-test when applicable. Nonparametric tests (Mann–Whitney or Kruskal–Wallis with Dunn’s post test) were used when data did not meet parametric assumptions. Analyses were conducted using InfoStat and GraphPad Prism 6. Differences were considered statistically significant at *p* < 0.05.

## Results

Considering the capacity of gene-based vaccines to elicit robust cellular immune responses, CruSEG DNA delivered by the attenuated *Salmonella enterica* serovar Typhimurium aroA strain SL7207 (Saro A) was evaluated as a vaccine candidate. Different immunization protocols were tested, including vaccination with CruSEG DNA alone or a heterologous prime–boost (P/B) strategy combining the *Salmonella*-based DNA delivery system with recombinant CruSEG formulated with CpG-ODN (rCruSEG + CpG). A control group was immunized with *Salmonella* carrying the empty plasmid (Saro A), and an additional group receiving rCruSEG + CpG was included for comparison. Female and male C3H/HeN mice were immunized either orally (o.) or intramuscularly (i.m.) four times at 10-day intervals, as detailed in **Table 1**.

**Table 1:** Mice immunizations.

Group/Dosis		1°	2°	3°	4°
GI	Saro A	Saro A o.	Saro A o.	Saro A o.	Saro A o.
GII	SCruSEG	SCruSEG o.	SCruSEG o.	SCruSEG o.	SCruSEG o.
GIII	rCruSEG+CpG	rCruSEG + CpG i.m	rCruSEG + CpG i.m.	rCruSEG + CpG i.m.	rCruSEG + CpG i.m.
GIV	Prime/Boost	SCruSEG o.	SCruSEG o.	rCruSEG + CpG i.m.	rCruSEG + CpG i.m.

O, Oral immunization; i.m., Intramuscular immunization.

To determine whether the different vaccination protocols induced functionally relevant immune responses, we first evaluated the antigen-specific humoral response after the last immunization.

### Antibody response

**Figure 1:**
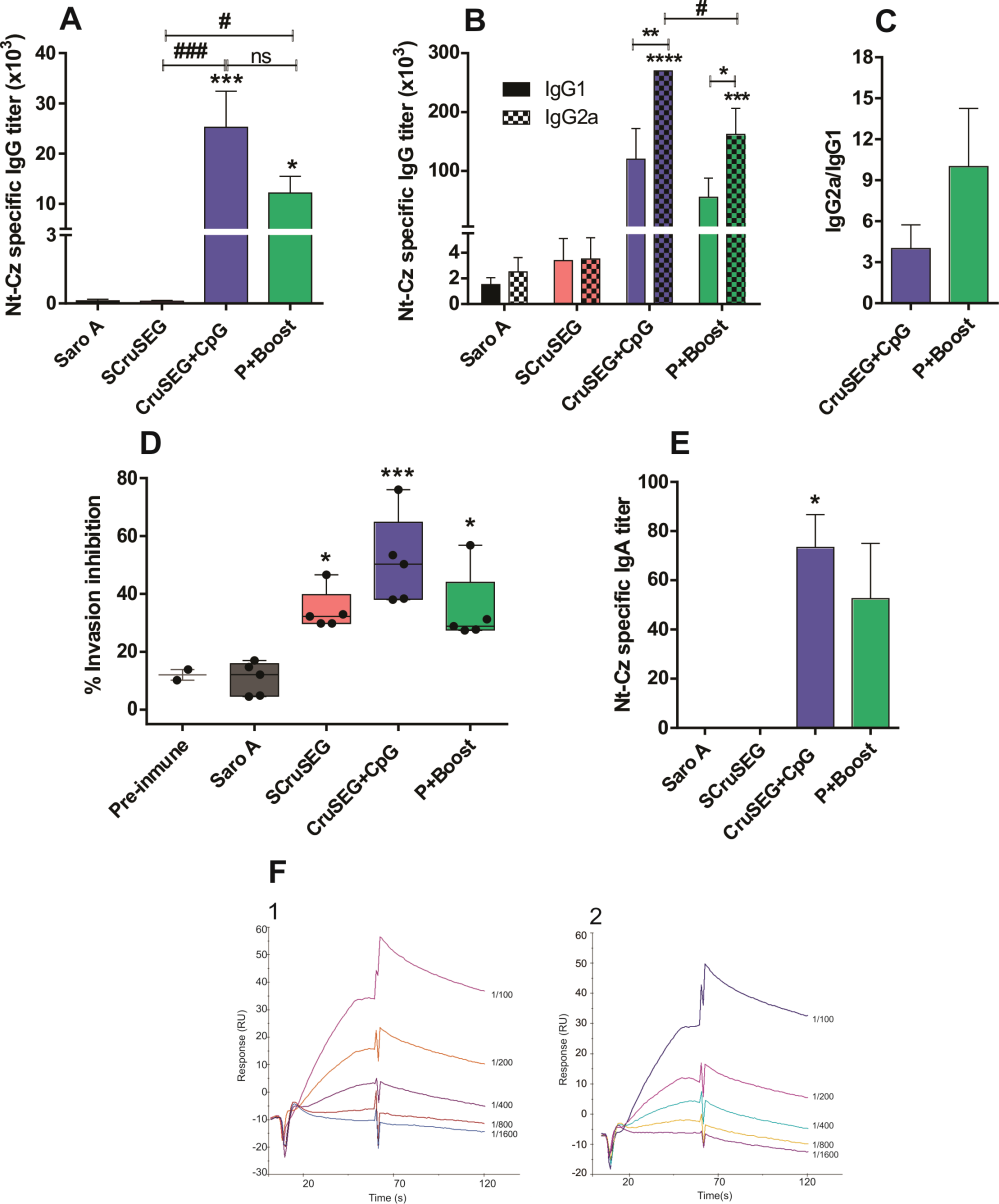
Antibody responses induced by different vaccination strategies. Serum samples were collected from 5 mice of each group 15 days after the last immunization, and antigen-specific antibody responses were analyzed. **(A)** Nt-Cz–specific IgG titers determined by indirect ELISA. **(B)** IgG isotype profile (IgG1 and IgG2a) specific for Nt-Cz. **(C)** IgG2a/IgG1 antibody ratio. **(D)** Inhibition of *Trypanosoma cruzi* infection of non-phagocytic Vero cells by immune sera. **(E)** Nt-Cz–specific IgA titers in sera from immunized mice. **(F)** Surface plasmon resonance (SPR) sensorgrams showing specific interactions between CruSEG and immune sera. CruSEG (30 μg/mL) was captured on a CM5 chip via immobilized anti-SEG IgG (6600 RU), followed by injection of serial dilutions of immune sera from the rCruSEG+CpG (F1) and SCruSEG (F2) groups; serum dilutions are indicated on the right. Comparison with Group 4 is shown in **Supplementary Figure 1**. Data represents the mean ± SEM of three independent experiments. Statistical analyses were performed using one-way ANOVA with Tukey’s *post hoc* test **(A, D)**, two-way ANOVA with Bonferroni’s *post hoc* test **(B)** or Kruskal–Wallis test with Dunn’s multiple comparison test **(E)**. Significant differences relative to the control group are indicated as follows: *p < 0.05; **p < 0.01; ***p < 0.001; ****p < 0.0001; ^#^p < 0.05; ^###^p < 0.001; ns, not significant.

The specific humoral response induced 15 days after the final immunization was analyzed in serum samples from vaccinated mice. Anti–N-terminal region of cruzipain (Nt-Cz) IgG titers were determined by ELISA. Mice immunized with at least two doses of rCruSEG+CpG developed high titers of Nt-Cz–specific IgG. In contrast, animals immunized with the four-dose SCruSEG regimen did not show a significant increase in antibody titers compared with the SaroA control group (**Figure 1A**). Analysis of IgG isotypes revealed that both the rCruSEG+CpG and prime–boost (P/B) groups developed significantly higher IgG2a than IgG1 titers (**Figure 1B**), consistent with a T helper 1 (Th1)-biased immune response. Notably, the P/B group exhibited an IgG2a/IgG1 ratio more than twofold higher than that observed in the rCruSEG+CpG group, indicating that priming with SCruSEG enhanced Th1 polarization (**Figure 1C**).

To evaluate the functional relevance of the elicited antibodies, a cell invasion inhibition assay was performed using *T. cruzi* Tulahuen strain trypomastigotes expressing *E. coli* β-galactosidase (Tul-β-gal). Sera from all immunized groups significantly inhibited parasite invasion of non-phagocytic Vero cells (**Figure 1D**). Remarkably, sera from the SCruSEG group, despite displaying low or undetectable Nt-Cz–specific IgG titers by ELISA, mediated more than 30% inhibition of parasite infection, suggesting the induction of functionally active antibody responses below the detection threshold of the assay.

To determine whether the neutralizing activity observed in sera from the SCruSEG group could be attributed to other antibody isotypes, Nt-Cz–specific IgA levels were evaluated in serum samples. As shown in **Figures 1E**, the IgA response profile closely mirrored that observed for IgG. Detectable Nt-Cz–specific IgA titers were observed exclusively in groups that received at least one dose of rCruSEG+CpG, although at substantially lower levels than IgG. In contrast, mice immunized with SCruSEG did not exhibit significant IgA titers. These results indicate that the neutralizing activity detected in SCruSEG immune sera could not be explained by the presence of Nt-Cz–specific IgA antibodies either.

Given these findings, a more sensitive approach was employed to investigate the presence of antigen-specific antibodies in SCruSEG sera. For this purpose, surface plasmon resonance (SPR) analysis was performed using a Biacore T100 biosensor and a capture-based assay. Polyclonal anti–staphylococcal superantigen G antibodies were purified and immobilized on a CM5 sensor chip. Recombinant CruSEG was subsequently captured via its SEGN24A domain, thereby exposing the Nt-Cz region for antibody recognition. Increasing dilutions of sera from the different immunization groups were then injected, and binding responses were recorded. A reference surface lacking immobilized ligand was used to control for nonspecific interactions.

As a positive control, sera from the rCruSEG+CpG group displayed typical sensorgrams characterized by rapid association and slow dissociation phases, indicative of specific antibody–antigen interactions (**Figure 1 F1**). Notably, sera from SCruSEG-immunized mice produced a comparable binding profile across the tested dilutions (**Figure 1 F2**). These results demonstrate the presence of specific interactions between SCruSEG immune sera and the chimeric antigen, suggesting that functionally relevant antibodies were induced but remained below the detection threshold of indirect ELISA.

Together, these findings indicate that rCruSEG+CpG and the prime–boost strategy elicited strong Nt-Cz–specific antibody responses with a clear Th1-biased isotype profile, consistent with a protective pattern against intracellular pathogens. In contrast, SCruSEG vaccination induced low levels of antigen-specific antibodies; however, these antibodies were functionally active, suggesting qualitative rather than purely quantitative differences in the humoral response.

Importantly, the magnitude of the antibody response did not fully correlate with the protection observed, indicating that humoral immunity alone cannot account for the protective effect. Therefore, we further evaluated the cell-mediated immune response, given its critical role in controlling intracellular protozoan infections and in mediating protection against *T. cruzi.*

### Analysis of the cellular immune response

**Figure 2:**
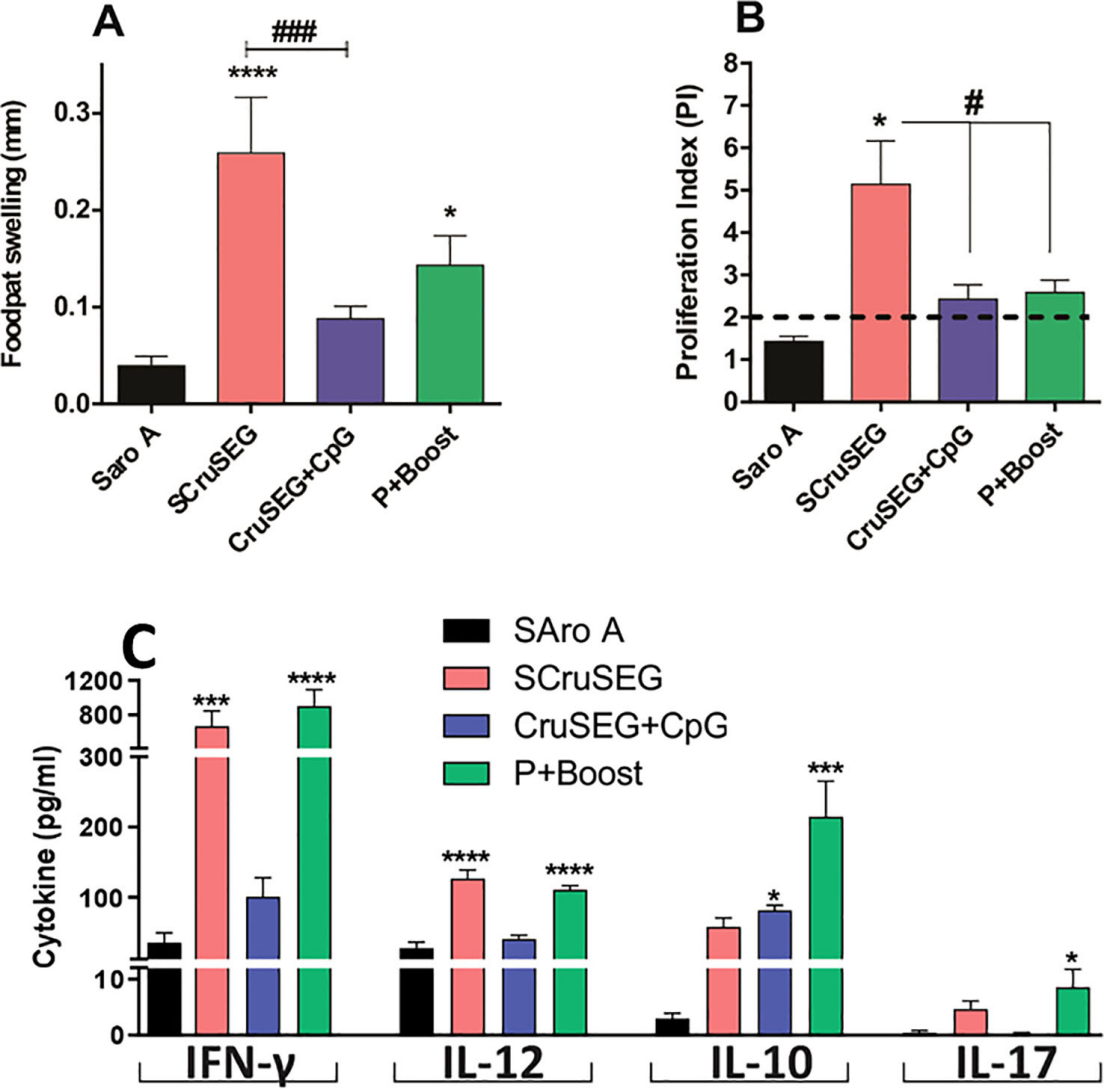
Analysis of the cellular immune response. **(A)** Delayed-type hypersensitivity (DTH) response. Results are expressed as the difference in footpad thickness measured before and 48 h after intradermal inoculation of recombinant Nt-Cz. **(B)** Antigen-specific *in vitro* proliferation following restimulation with recombinant Nt-Cz. Splenocytes from immunized mice were stimulated with 10 μg of rNt-Cz, and cell proliferation was assessed at 96 h by [³H]-thymidine incorporation. Results are expressed as the proliferation index (PI), calculated as the ratio of counts per minute (cpm) in the presence of rNt-Cz to cpm in the absence of antigen. **(C)** Cytokine secretion by splenocytes restimulated with rNt-Cz. Cytokine levels in culture supernatants were quantified by capture ELISA 48 h after *in vitro* restimulation with 10 μg of rNt-Cz. Bars represent the mean ± SEM of three independent experiments. Statistical analyses were performed using one-way ANOVA with Tukey’s *post hoc* test **(A, C)** or Kruskal–Wallis test with Dunn’s multiple comparison test **(B)**. Significant differences relative to the Saro A control group are indicated as follows: *p < 0.05; ***p < 0.001; ****p < 0.0001; ^#^p < 0.05; ^###^p < 0.001.

To evaluate the *in vivo* cellular immune response induced by the different vaccination strategies, a delayed-type hypersensitivity (DTH) assay was performed 15 days after the last immunization. Footpad thickness was measured before and 48 h after intradermal inoculation of 10 µg of recombinant Nt-Cz. All immunized groups developed antigen-specific cellular responses significantly higher than those observed in the SaroA control group. Notably, responses were significantly stronger in groups that received at least two doses of SCruSEG, with the group immunized with four doses of SCruSEG exhibiting the most robust DTH response (*p* < 0.0001) (**Figure 2A**).

To further characterize antigen-specific cellular immunity, splenocytes were isolated from immunized mice 15 days after the final dose and the proliferative capacity was evaluated by [³H]-thymidine incorporation. In agreement with the *in vivo* DTH results, splenocytes from the SCruSEG four-dose group showed the highest antigen-specific proliferative response, which was significantly greater than that of the SaroA control group (*p* < 0.05) (**Figure 2B**). Although increased proliferation was also observed in the rCruSEG+CpG and prime–boost (P/B) groups, these differences did not reach statistical significance compared with the control.

To assess the functional profile of the T cell response, cytokine secretion was analyzed in culture supernatants of splenocytes stimulated *in vitro* with recombinant Nt-Cz. Levels of IFN-γ, IL-12, IL-10, and IL-17 were quantified by ELISA. All immunized groups secreted IFN-γ, IL-12, and IL-10 (**Figure 2C**). Importantly, IFN-γ and IL-12 secretion profiles aligned with the results obtained in the DTH and proliferation assays, with significantly higher levels detected in groups that received at least two doses of SCruSEG compared with the SaroA control group (**Figure 2C**). Analysis of IL-10 secretion revealed detectable levels in all groups; however, only the prime–boost group showed a statistically significant increase relative to the control.

In addition, IL-17 secretion, a hallmark cytokine of the Th17 response with a reported protective role in *T. cruzi* infection (16, 29, 30), was evaluated. Although overall IL-17 levels were low, the highest concentrations were detected in the prime–boost group (**Figure 2C**).

### CD4^+^ T cell response

**Figure 3:**
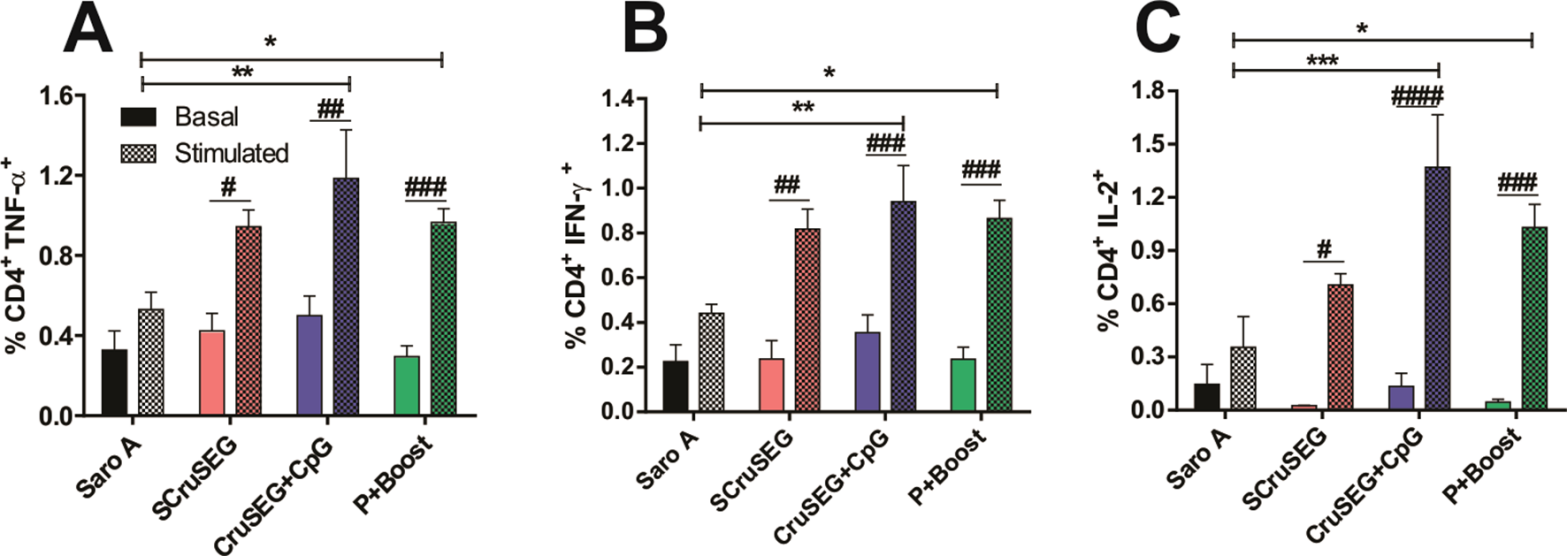
Intracellular cytokine production by CD4^+^ T cells. Percentages of CD4^+^ T cells producing TNF-α **(A)**, IFN-γ **(B)**, and IL-2 **(C)** following *in vitro* restimulation with recombinant Nt-Cz. Statistical analysis was performed using two-way ANOVA followed by Tukey’s *post hoc* test. Significant differences relative to the Saro A control group are indicated as follows: *p < 0.05; **p < 0.01; ***p < 0.001; ^#^p < 0.05; ^##^p < 0.01; ^###^p < 0.001; ^####^p < 0.0001.

To further characterize the cellular immune response induced by the different vaccination protocols, intracellular cytokine production by CD4^+^ T cells was analyzed by flow cytometry following *in vitro* stimulation of splenocytes with recombinant Nt-Cz. The frequencies of CD4^+^ T cells producing TNF-α, IFN-γ, and IL-2 were assessed. All immunized groups displayed increased frequencies of TNF-α^+^, IFN-γ^+^, and IL-2^+^ CD4^+^ T cells compared with non-stimulated controls. Notably, upon antigenic stimulation, only the rCruSEG+CpG and prime–boost (P/B) groups showed significantly higher frequencies of cytokine-producing CD4^+^ T cells compared with the SaroA control group (**Figures 3A–C**).

**Figure 4:**
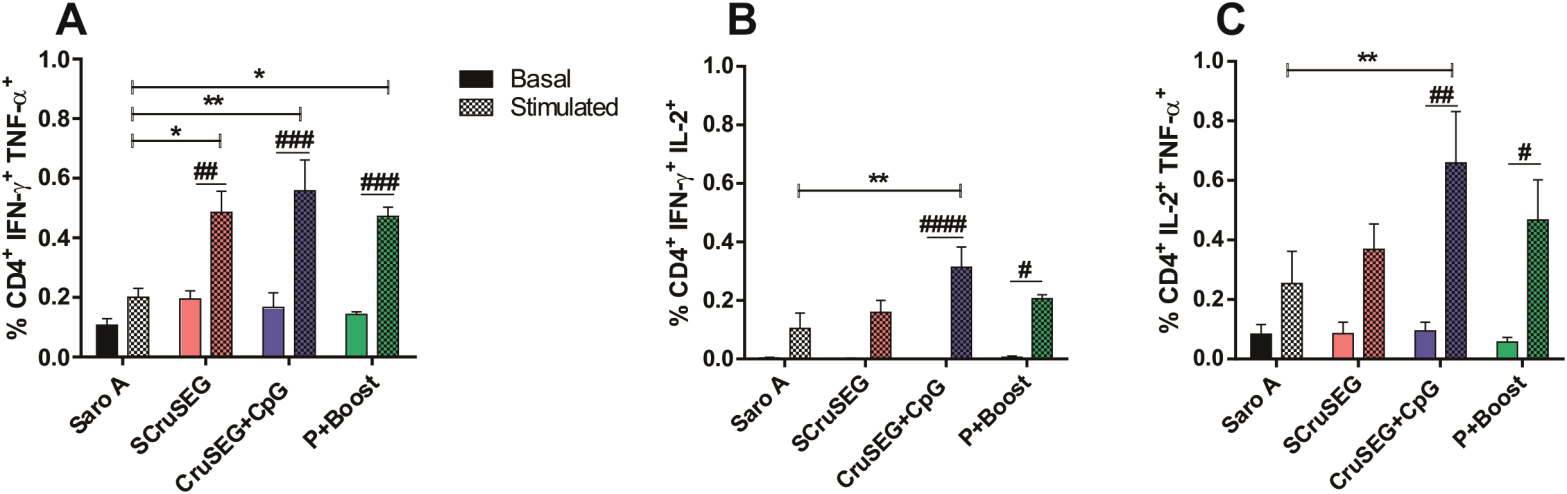
Induction of dual-cytokine–producing CD4^+^ T cell subsets. Percentages of CD4^+^ T cells simultaneously producing two cytokines: IFN-γ^+^ TNF-α^+^**(A)**, TNF-α^+^ IL-2^+^**(B)**, and IFN-γ^+^ IL-2^+^**(C)**, following *in vitro* restimulation with recombinant Nt-Cz. Statistical analysis was performed using two-way ANOVA followed by Tukey’s *post hoc* test. Significant differences relative to the SaroA control group are indicated as follows: *p < 0.05; **p < 0.01; ^#^p < 0.05; ^##^p < 0.01; ^###^p < 0.001; ^####^p < 0.0001.

The polyfunctionality of CD4^+^ T cells, defined as the capacity to simultaneously produce two or more cytokines (31), was also evaluated. As shown in **Figure 4A**, the frequency of CD4^+^ T cells co-producing IFN-γ and TNF-α was significantly higher in all immunized groups following antigenic stimulation compared with the SaroA control group. In addition, increased frequencies of CD4^+^ TNF-α^+^ IL-2^+^ and CD4^+^ IFN-γ^+^ IL-2^+^ T cells were detected in all vaccinated groups, with the highest levels observed in mice immunized with rCruSEG+CpG (**Figures 4B, C**). Notably, this polyfunctional profile was exclusively associated with immunized animals, as splenocytes from control mice failed to exhibit simultaneous cytokine production upon Nt-Cz restimulation.

**Figure 5:**
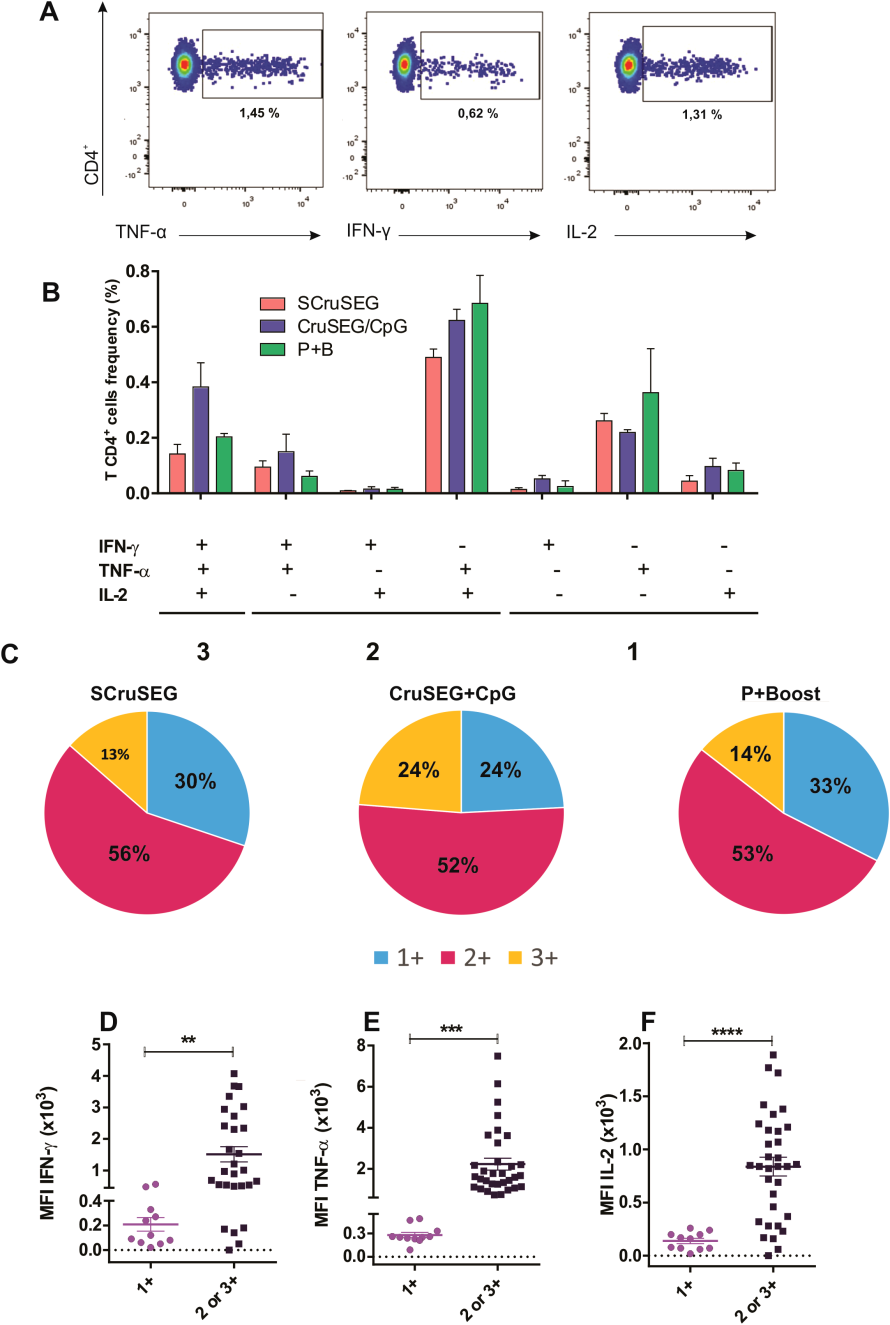
Functional profile and quality of polyfunctional CD4^+^ T cells. **(A)** Boolean gating strategy used to determine the simultaneous production of IFN-γ, TNF-α, and IL-2 by CD4^+^ T cells. **(B)** Frequencies of CD4^+^ T cells producing IFN-γ, TNF-α, and IL-2 in each of the seven possible cytokine combinations. **(C)** Distribution of CD4^+^ T cells producing three (3^+^), two (2^+^), or one (1^+^) cytokine. **(D)** Mean fluorescence intensity (MFI) of IFN-γ, TNF-α, and IL-2 in monofunctional (1^+^) and polyfunctional (2^+^ or 3^+^) CD4^+^ T cell populations. Statistical analysis was performed using Student’s *t*-test. **p < 0.01; ***p < 0.001; ****p < 0.0001.

To further characterize the polyfunctionality of Nt-Cz–specific CD4^+^ T cells, a gating strategy was applied to assess the simultaneous production of not only two, but also all three cytokines analyzed (**Figure 5A**). As shown in **Figure 5B**, all immunized groups exhibited higher frequencies of CD4^+^ T cells producing two or three cytokines concurrently compared with cells producing a single cytokine. Accordingly, polyfunctional CD4^+^ T cells consistently predominated over monofunctional cells across all vaccination protocols (**Figure 5C**), accounting for 69–76% versus 24–33% of the total cytokine-producing CD4^+^ T cell population, respectively. Notably, the rCruSEG+CpG group displayed the highest frequency of triple-positive IFN-γ^+^ TNF-α^+^ IL-2^+^ CD4^+^ T cells.

In addition to their increased frequency, polyfunctional CD4^+^ T cells exhibited significantly higher per-cell production levels of IFN-γ, TNF-α, and IL-2 compared with monofunctional cells, as reflected by the increased median fluorescence intensity (MFI) values shown in **Figure 5D**.

To further dissect the complexity and functional quality of the CD4^+^ T cell response, we next analyzed the distribution of double- and triple-cytokine–producing cells, as well as the magnitude of cytokine production at the single-cell level.

**Figure 6:**
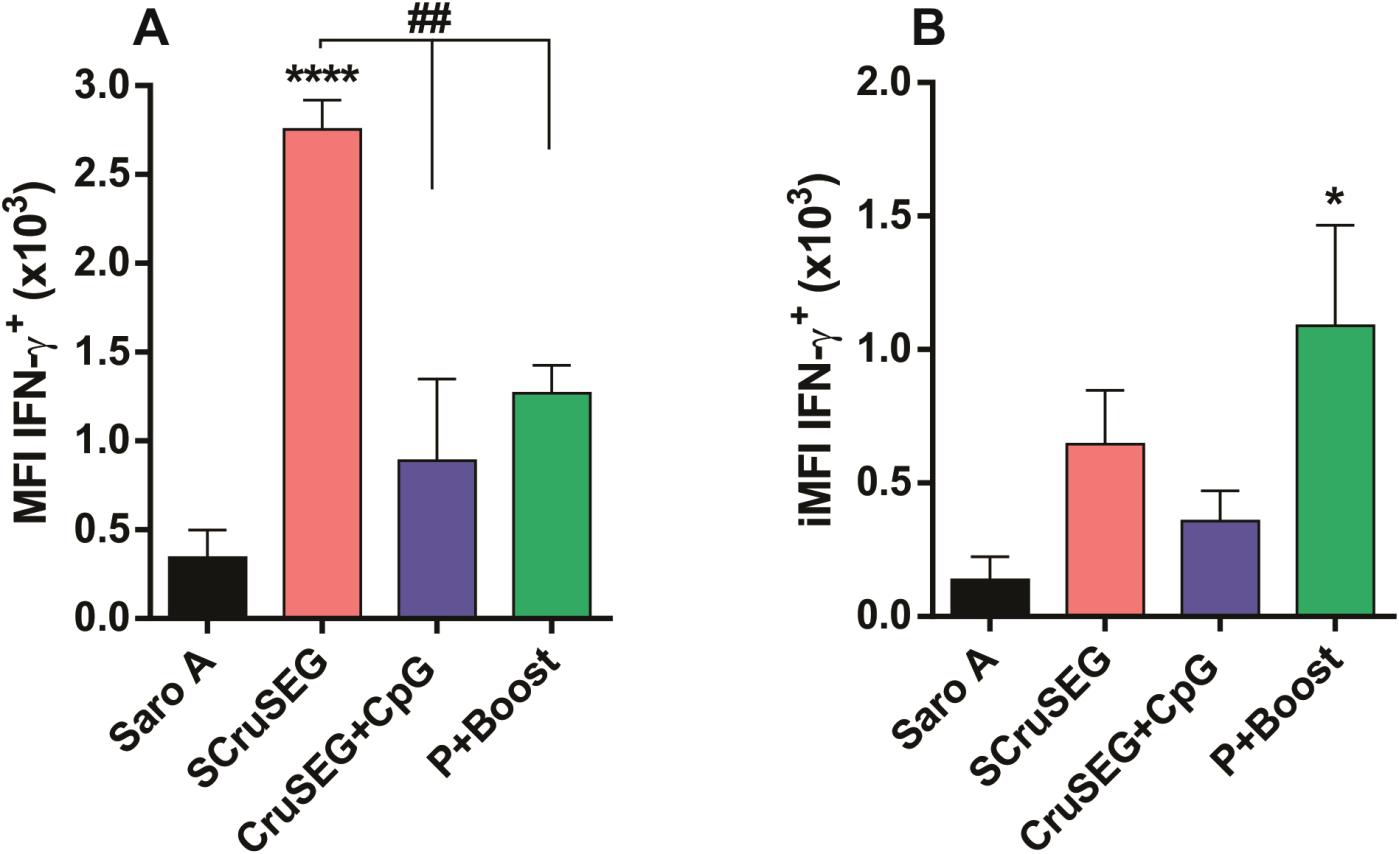
MFI and iMFI parameters of IFN-γ production by CD4^+^ T cells. **(A)** Mean fluorescence intensity (MFI) of IFN-γ in monofunctional and polyfunctional CD4^+^ T cells for each immunized group. **(B)** Integrated mean fluorescence intensity (iMFI) for IFN-γ, calculated as the product of the percentage of IFN-γ–producing CD4^+^ T cells and the corresponding MFI value for each immunized group. Statistical analysis was performed using one-way ANOVA followed by Tukey’s *post hoc* test. Significant differences relative to the SaroA control group are indicated as follows: *p < 0.05; ****p < 0.0001; ^##^p < 0.01.

Given that the intracellular frequency of IFN-γ–producing CD4^+^ T cells did not fully correlate with IFN-γ levels measured in culture supernatants by ELISA, mean fluorescence intensity (MFI) was analyzed to assess cytokine production at the single-cell level. As shown in **Figure 6A**, comparison of IFN-γ MFI values in CD4^+^ T cells—independent of whether cells produced one or multiple cytokines—revealed that the SCruSEG group exhibited the highest per-cell IFN-γ production.

In addition, the integrated mean fluorescence intensity (iMFI) was calculated to better estimate the overall magnitude of intracellular cytokine responses by integrating both the frequency of cytokine-producing cells and their MFI values (32). Notably, the iMFI pattern for IFN-γ across immunized groups were consistent with the levels of IFN-γ detected in culture supernatants by ELISA. In this analysis, groups immunized with at least two doses of SCruSEG displayed the highest overall IFN-γ responses (**Figure 6B**).

Together, these data indicate distinct qualitative patterns of IFN-γ production among vaccination strategies: rCruSEG+CpG immunization was associated with activation of a larger proportion of IFN-γ–producing CD4^+^ T cells, whereas SCruSEG and prime–boost regimens induced a smaller population of CD4^+^ T cells with higher per-cell IFN-γ production, resulting in increased cumulative cytokine release.

### CD8^+^ T cell response

Given the central role of CD8^+^ T cells in the control of *Trypanosoma cruzi* infection through the elimination of infected cells (33), CD8^+^ T cell functionality was evaluated by flow cytometry. Splenocytes from immunized mice were stimulated *in vitro* with recombinant Nt-Cz together with the Nt-Cz–derived peptide KEEASSAVV, which displays a high predicted binding affinity for MHC class I molecules of the H-2K^k^ haplotype (C3H/HeN) and is therefore suitable for CD8^+^ T cell activation.

**Figure 7:**
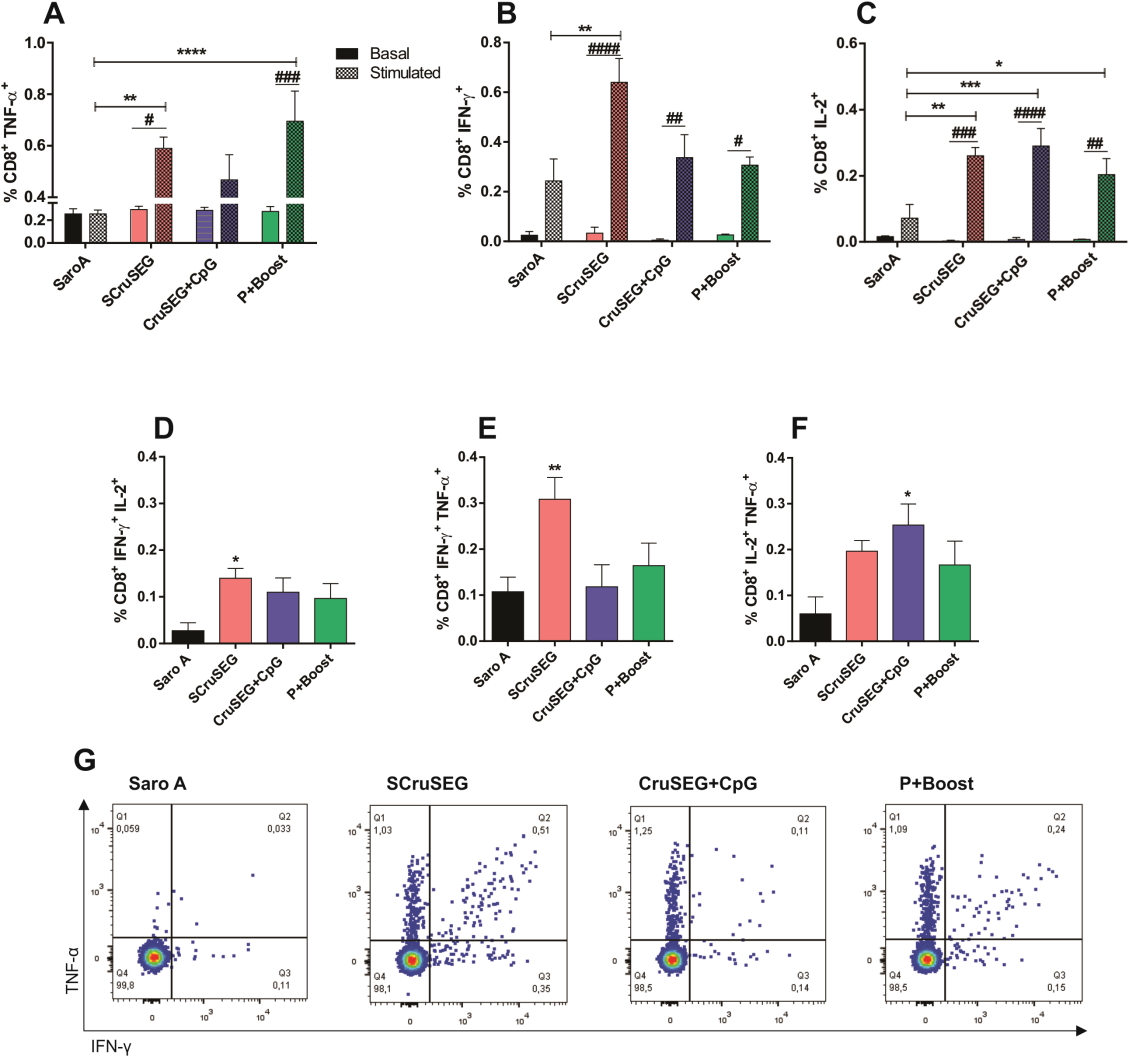
Functional profile of the CD8^+^ T cell response. Percentages of CD8^+^ T cells producing TNF-α **(A)**, IFN-γ **(B)**, and IL-2 **(C)** following *in vitro* restimulation with recombinant Nt-Cz and the Nt-Cz–derived peptide KEEASSAVV. **(D–F)** Percentages of dual cytokine–producing CD8^+^ T cells: IFN-γ^+^ TNF-α^+^**(D)**, IFN-γ^+^ IL-2^+^**(E)**, and IL-2^+^ TNF-α^+^**(F, G)** Representative flow cytometry dot plots of dual IFN-γ^+^ TNF-α^+^ CD8^+^ T cells. Statistical analysis was performed using two-way ANOVA followed by Tukey’s *post hoc* test for panels **(A–C)**, or one-way ANOVA with Tukey’s *post hoc* test for panels **(D–F)**. Significant differences relative to the Saro A control group are indicated as follows: *p < 0.05; **p < 0.01; ***p < 0.001; ****p < 0.0001; ^#^p < 0.05; ^##^p < 0.01; ^###^p < 0.001; ^####^p < 0.0001.

Analysis of intracellular cytokine production revealed that CD8^+^ T cells from mice immunized with at least two doses of SCruSEG exhibited the highest frequencies of IFN-γ^+^, TNF-α^+^, and IL-2^+^ cells, with significant differences compared with the SaroA control group (**Figures 7A–C**). Notably, this response pattern differed from that observed in the CD4^+^ T cell compartment. In particular, the SCruSEG group was the only vaccination group that showed a significant increase in IFN-γ production by CD8^+^ T cells upon antigenic stimulation (**Figure 7B**).

When CD8^+^ T cell polyfunctionality was evaluated, the pattern of dual cytokine–producing cells closely mirrored that observed for individual cytokines. Consistent with previous analyses, the SCruSEG group exhibited significantly higher frequencies of CD8^+^ T cells co-producing IFN-γ and TNF-α, as well as IFN-γ and IL-2, compared with the control group (**Figures 7D–G**). Notably, in all cases, the frequencies of cytokine-producing CD8^+^ T cells following *in vitro* restimulation were lower than those observed in the CD4^+^ T cell compartment.

**Figure 8:**
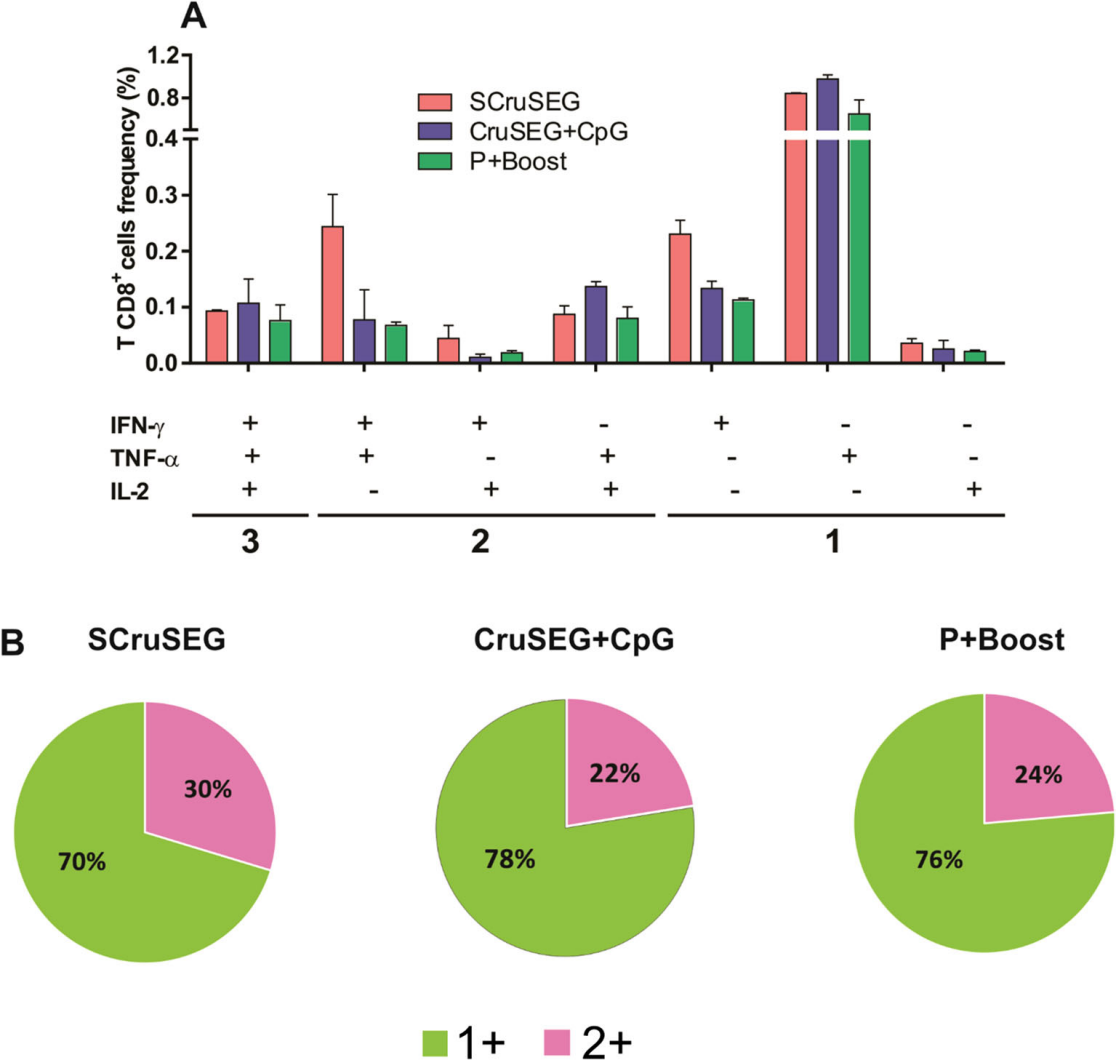
Polyfunctionality of the CD8^+^ T cell response. **(A)** Frequencies of CD8^+^ T cells producing IFN-γ, TNF-α, and IL-2 in each of the seven possible cytokine combinations following antigenic restimulation. **(B)** Pie charts showing the distribution of monofunctional CD8^+^ T cells producing one cytokine (1^+^) and polyfunctional cells producing 2 cytokines.

CD8^+^ T cell polyfunctionality was then further characterized using an approach analogous to that applied for CD4^+^ T cells. In contrast to the CD4^+^ compartment, immunized groups displayed a predominance of monofunctional over polyfunctional CD8^+^ T cells (**Figure 8A**). This trend was consistent across all vaccination protocols, with monofunctional cells accounting for 70–78% and polyfunctional cells for 22–30% of cytokine-producing CD8^+^ T cells (**Figure 8B**). Despite this overall pattern, the SCruSEG group developed the highest proportion of polyfunctional CD8^+^ T cells among the immunized groups.

### Efficacy of vaccine candidates after *Trypanosoma cruzi* challenge

To evaluate the protective efficacy conferred by the different vaccination protocols, mice were challenged with *T. cruzi* trypomastigotes as a proof-of-concept approach. Protection was assessed during the acute phase of infection, the extent of infection during the chronic phase, and parameters indicative of tissue damage.

### Protection during acute phase of infection with a highly virulent *T. cruzi* strain

To assess protection during the acute phase of infection, female C3H/HeN mice were challenged intraperitoneally with 1,000 bloodstream trypomastigotes of the highly virulent RA strain two weeks after the last immunization. Parasitemia and body weight were monitored every 2–3 days throughout the acute phase.

**Figure 9:**
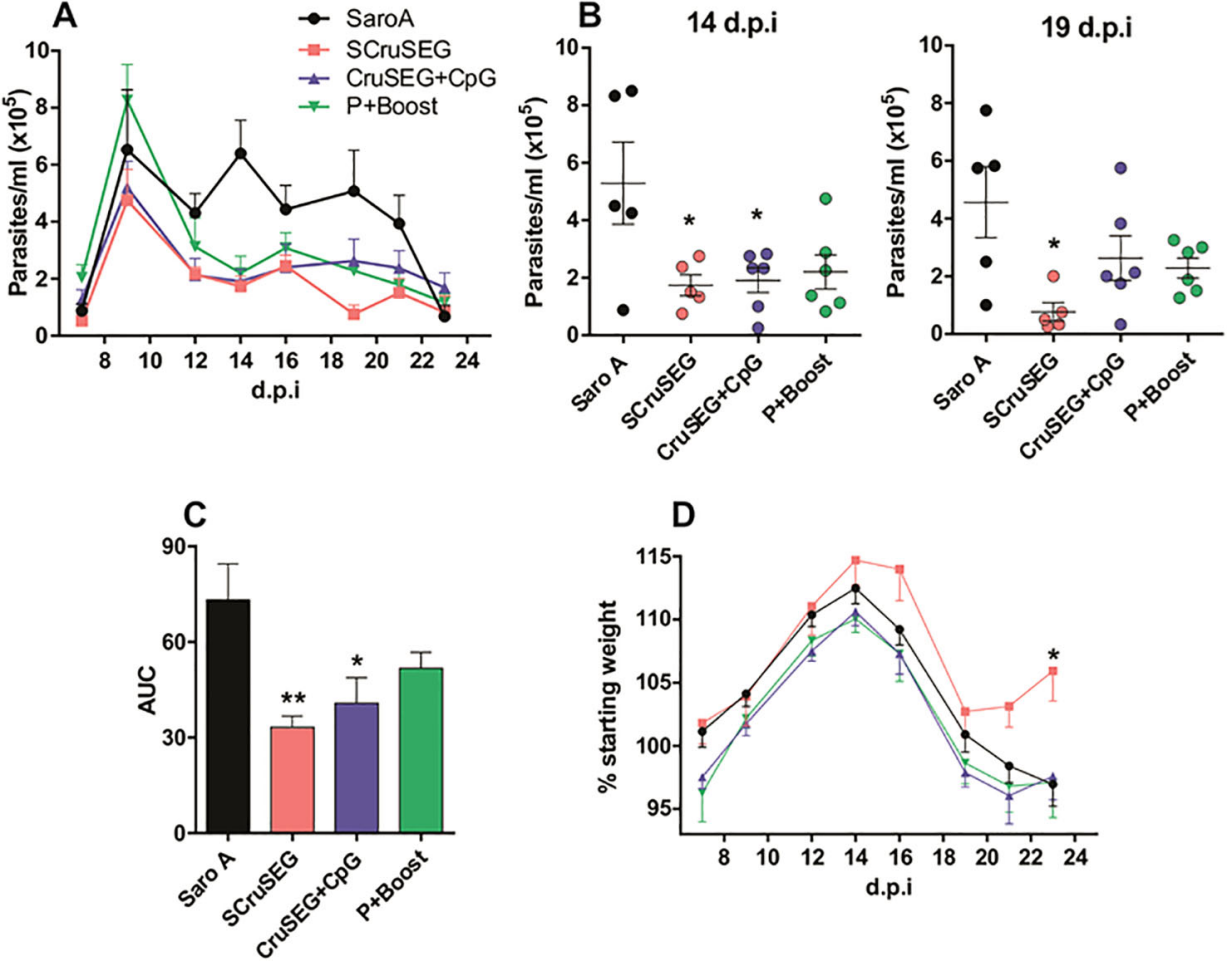
Protection against challenge with *T. cruzi* trypomastigotes of the RA strain. Two weeks after the final immunization,5 mice from groups 1 and 2 and 6 mice from groups 4 and 5, were challenged with bloodstream trypomastigotes of the highly virulent RA strain. **(A)** Parasitemia monitored throughout the acut phase of infection. **(B)** Circulating parasite levels at the peak of the acute phase (14 dpi; left) and at 19 dpi (right). **(C)** Area under the parasitemia curve (AUC) calculated for the entire acute phase. **(D)** Body weight variation during the acute phase, expressed as the percentage of each mouse’s weight relative to its pre-infection value. Statistical analysis was performed using one-way ANOVA followed by Tukey’s *post hoc* test **(B, C)**. Significant differences are indicated as follows: *p < 0.05; **p < 0.01.

All immunized groups exhibited a marked reduction in parasite burden during the acute phase compared with the SaroA control group (**Figure 9A**). This effect was particularly pronounced in the SCruSEG and rCruSEG+CpG groups at the peak of parasitemia (14 dpi). At later stages of the acute phase (19 dpi), the SCruSEG group showed superior control of circulating parasite levels (**Figure 9B**). Consistently, analysis of the cumulative parasite burden across the entire acute phase revealed a significant reduction in the area under the curve (AUC) in both the SCruSEG and rCruSEG+CpG groups compared with controls (**Figure 9C**).

In addition to parasitemia control, mice immunized with SCruSEG exhibited the lowest degree of weight loss throughout the acute phase, which was significantly reduced relative to the other experimental groups. Notably, these mice even displayed weight gain as parasitemia became controlled around day 18 post-infection (**Figure 9D**).

### Protection during the chronic phase of infection with a low virulence *T. cruzi* strain

Although control of parasitemia during the acute phase is critical, sustained protection during chronic infection is essential, as this phase is characterized by parasite persistence in target tissues and the development of Chagas pathology. To evaluate vaccine efficacy in this context, infection with the myotropic K98 clone, derived from the CA-I strain (DTU TcI), was used as a model of chronic *T. cruzi* infection.

Male C3H/HeN mice were immunized and, fifteen days after the final dose, challenged with a sublethal inoculum of K98 bloodstream trypomastigotes. Parasitemia was monitored weekly during the acute phase of infection.

**Figure 10:**
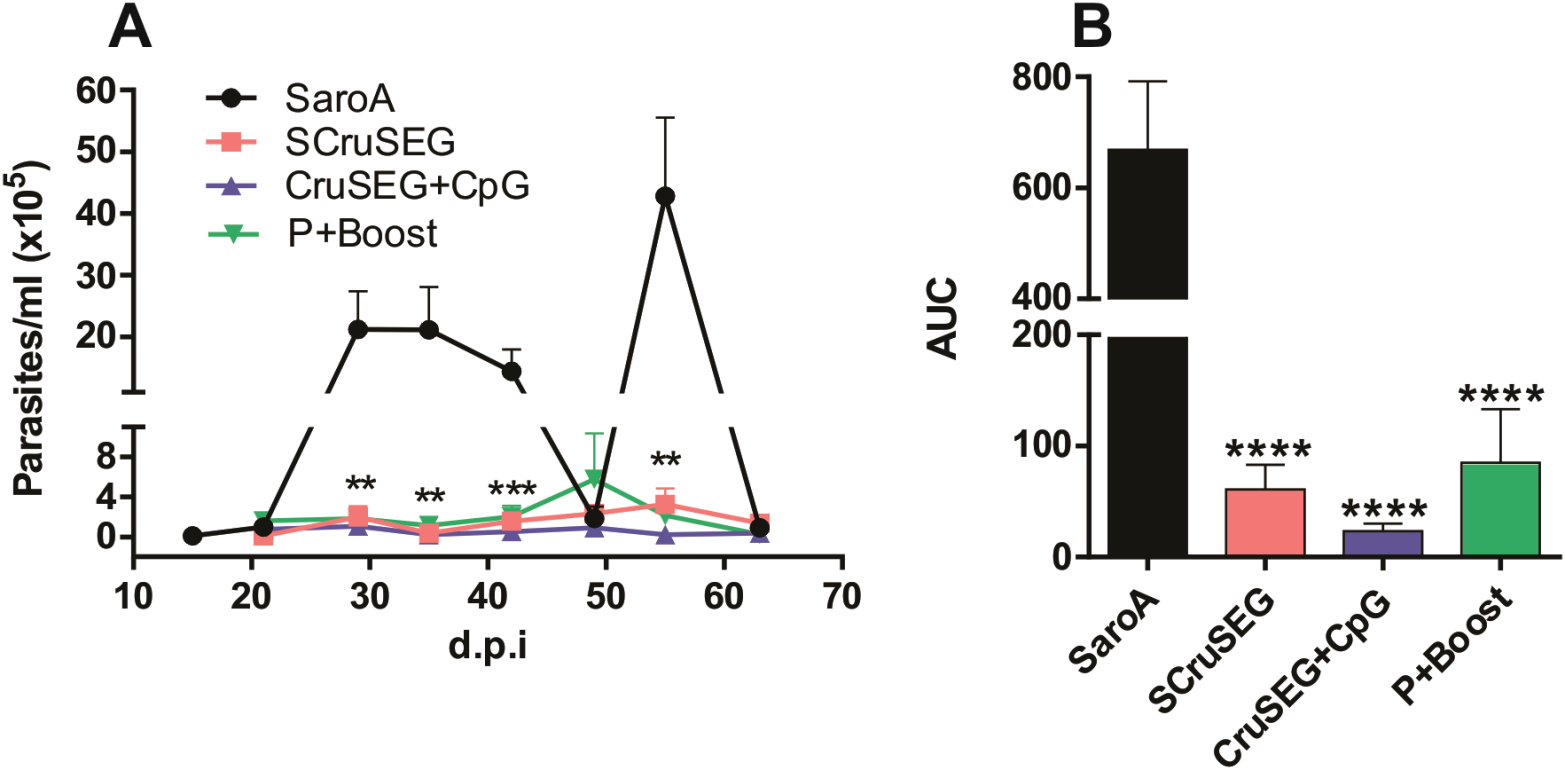
Protection during acute infection with the myotropic *Trypanosoma cruzi* K98 clone. Two weeks after the final immunization, mice were challenged intraperitoneally with a sublethal dose of 3 × 10^5^ bloodstream trypomastigotes of the K98 clone. **(A)** Parasitemia monitored weekly during the acute phase of infection. **(B)** Area under the parasitemia curve (AUC) calculated for the acute phase. Statistical analysis was performed using one-way ANOVA followed by Tukey’s *post hoc* test. **p < 0.01; ***p < 0.001; ****p < 0.0001.

All immunized groups exhibited a marked reduction in parasitemia during the acute phase compared with the SaroA control group (**Figure 10A**). Consistently, total parasite burden during acute infection was significantly reduced in vaccinated mice, with decreases in the parasitemia area under the curve ranging from 9- to 29-fold relative to controls (**Figure 10B**). These results demonstrate the capacity of the vaccination protocols to control acute *T. cruzi* infection caused by parasite strains belonging to distinct DTUs.

### Evaluation of tissue damage markers

**Figure 11:**
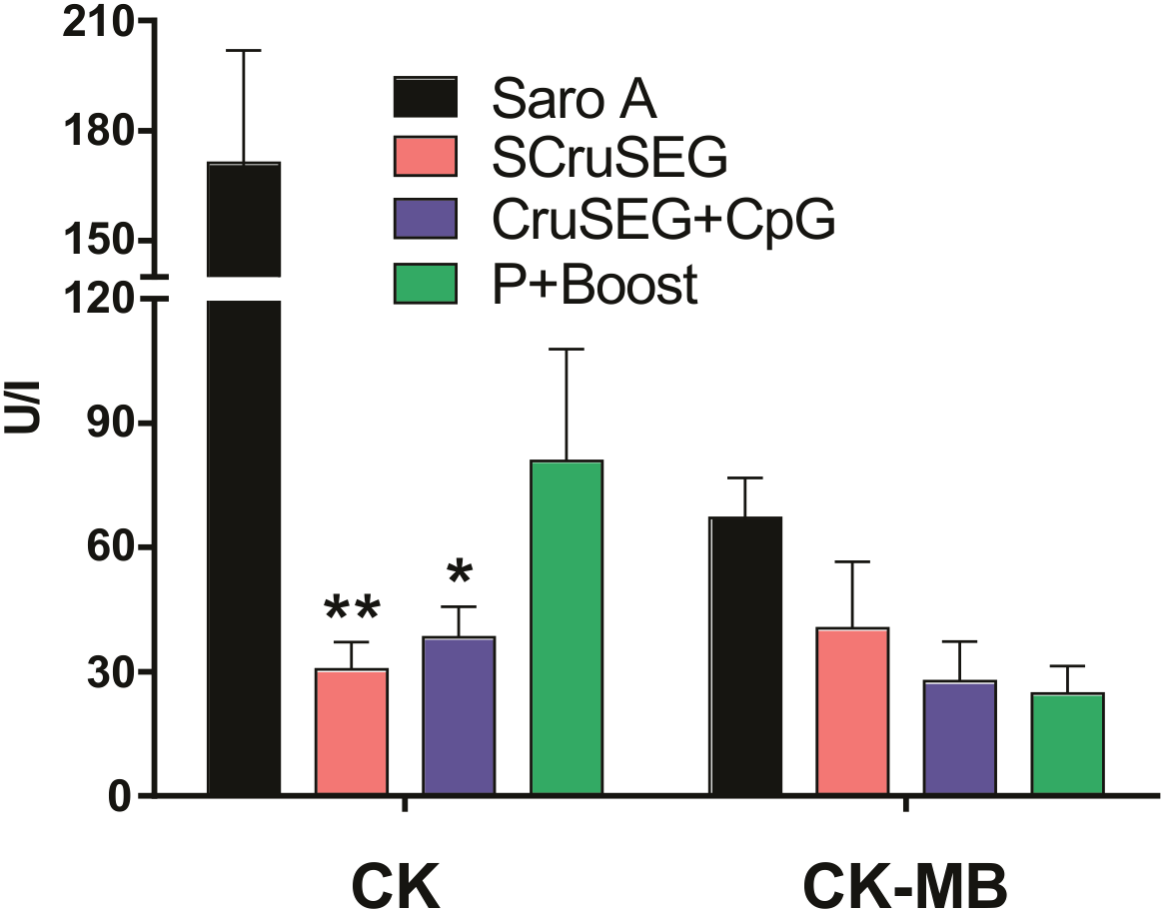
Serum levels of CK and CK-MB at 120 dpi. Serum activity of creatine kinase (CK) and its cardiac-specific isoenzyme (CK-MB) were measured in immunized and challenged mice at 120 days dpi. Bars represent the mean ± SEM of two independent experiments. Statistical analysis was performed using the Kruskal–Wallis test followed by Dunn’s multiple comparison test. Significant differences are indicated as follows: *p < 0.05; **p < 0.01.

During the chronic phase of infection, parameters indicative of tissue damage were evaluated. Serum levels of creatine kinase (CK) and its cardiac isoenzyme (CK-MB) were measured at 120 dpi. All immunized groups exhibited markedly reduced levels of both enzymes compared with infected, non-vaccinated control animals (**Figure 11**). Notably, mice immunized with SCruSEG or rCruSEG+CpG showed statistically significant reductions in total CK activity relative to the control group, indicating decreased muscle tissue damage.

To corroborate the reduced tissue damage indicated by serum enzyme activity, K98-infected immunized mice were euthanized at 120 dpi, and skeletal and cardiac muscle tissues were collected for histopathological analysis. Tissue sections were prepared and stained with hematoxylin and eosin (H&E).

**Figure 12:**
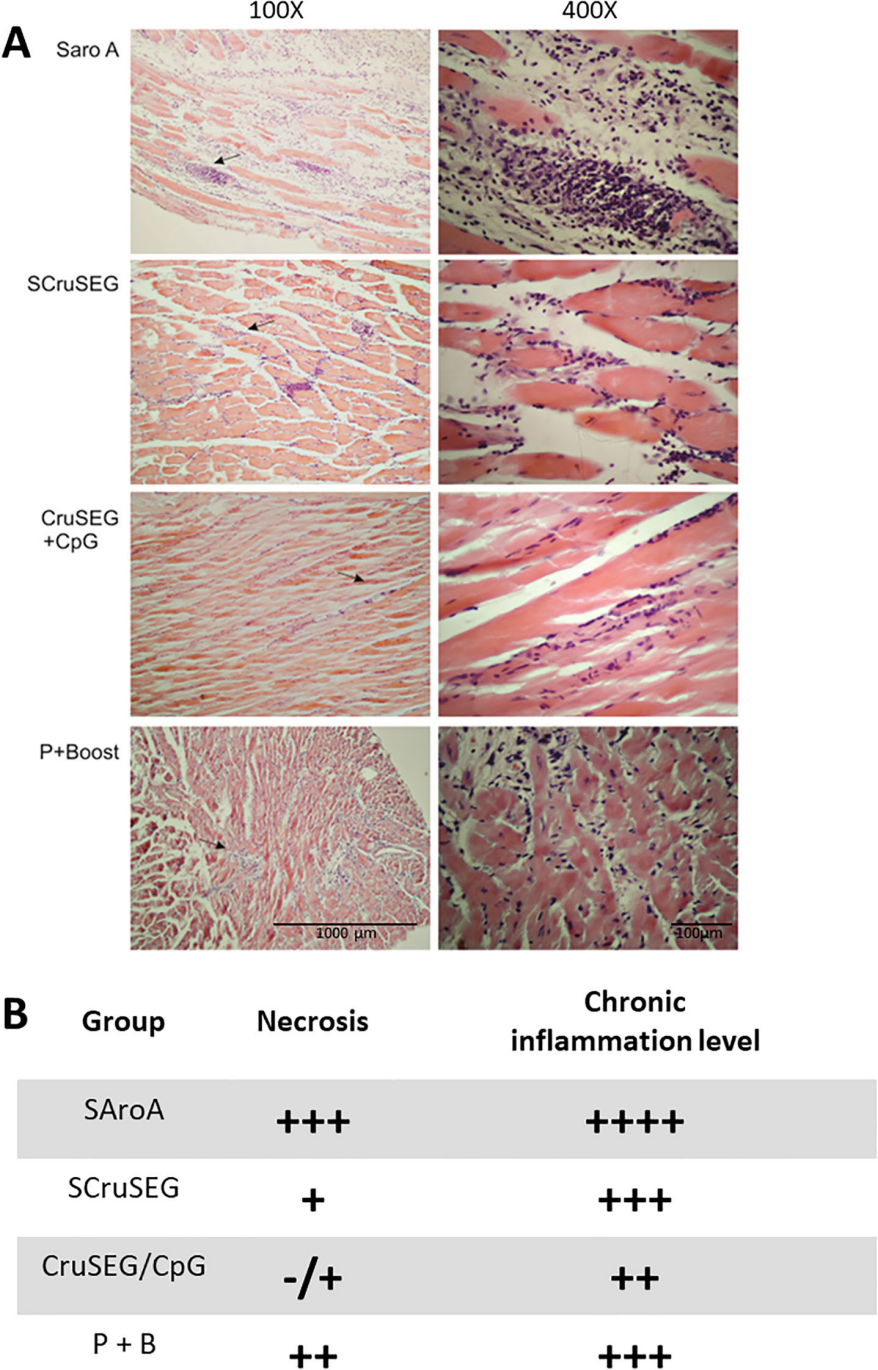
Histopathological analysis of skeletal muscle tissue at 120 dpi. Representative hematoxylin and eosin–stained sections of skeletal muscle from immunized mice during the chronic phase of K98 infection. **(A)** Photomicrographs obtained at 100× (left) and 400× (right) magnification. **(B)** Semi-quantitative assessment of tissue damage. The extent of inflammatory infiltrate was graded as follows: +, isolated foci; ++, multiple isolated foci; +++, multiple confluent foci; ++++, diffuse inflammation throughout the section. Myofiber necrosis was scored according to its frequency: −/+, absent or scarce; +, sparse; ++, intermediate; +++, extensive. Arrows in the 100× images indicate the areas shown at higher magnification (400×).

Histological examination of skeletal muscle revealed lesions consistent with a chronic inflammatory process, characterized by lymphocytic infiltrates of varying intensity. Tissue damage was evaluated using a semi-quantitative scoring system, as described in the legend to **Figure 12**. The non-immunized control group exhibited the most severe pathology, with multiple confluent and non-confluent inflammatory foci, extensive myocyte necrosis, and, in some sections, inflammatory infiltration extending into adjacent adipose tissue (**Figure 12**, upper panel). In contrast, skeletal muscle sections from immunized mice displayed milder lesions, with necrosis restricted to limited areas and predominantly isolated, non-confluent inflammatory foci. The most pronounced reductions in tissue damage were observed in the CruSEG+CpG and SCruSEG groups, consistent with the decreased CK enzyme levels measured in these animals (**Figure 12**).

**Figure 13:**
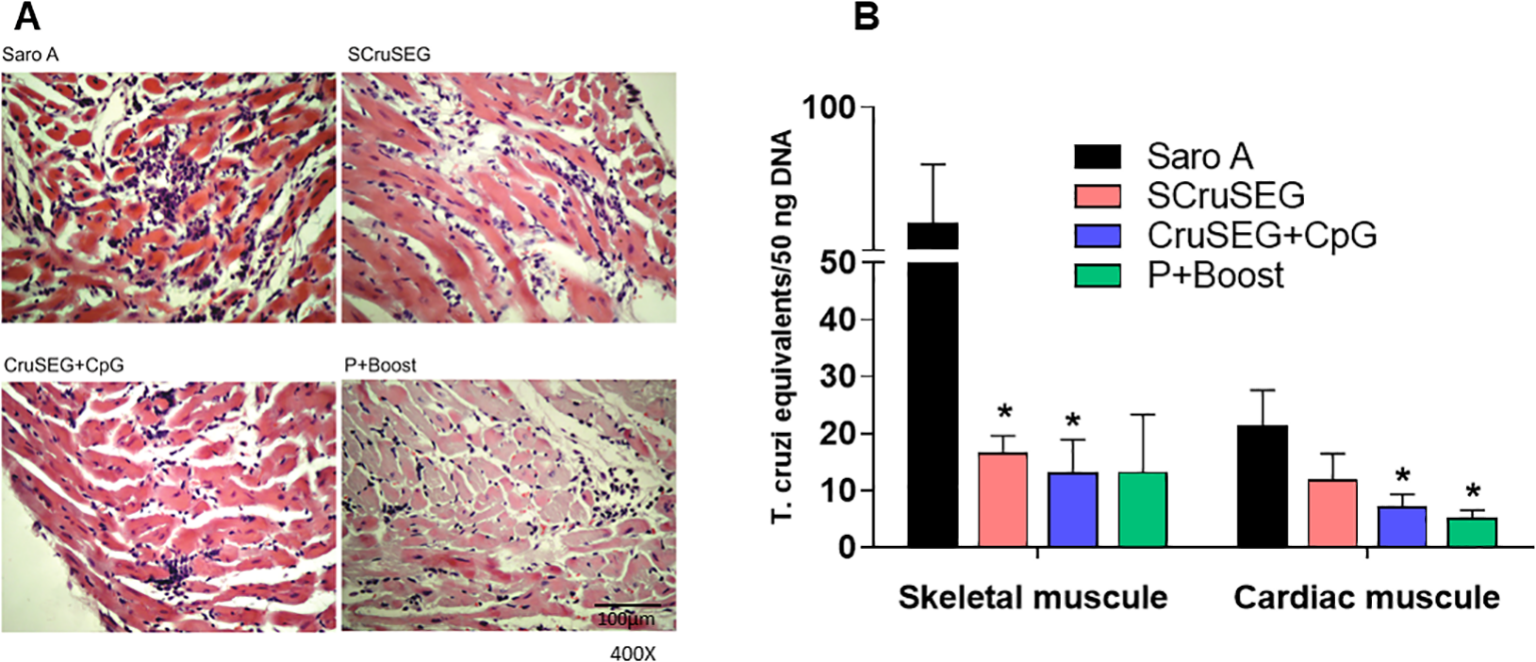
Protection during the chronic phase of *Trypanosoma cruzi* infection. **(A)** Representative histological sections of cardiac muscle from K98-infected mice at 120 days post-infection, showing mononuclear inflammatory infiltrates in control and immunized groups (H&E staining). **(B)** Parasite burden in skeletal and cardiac muscle quantified by real-time PCR at 120 days post-infection. Data represents parasite equivalents normalized to host DNA. Bars represent mean ± SEM. Statistical significance is indicated relative to the control group. *p < 0.05.

When cardiac muscle tissues were analyzed, overall damage was lower than that observed in skeletal muscle, consistent with the myotropic profile of the CA-I strain. Nevertheless, differences were detected in the degree of mononuclear inflammatory infiltrate, with the non-immunized control group displaying a higher number of confluent and non-confluent inflammatory foci compared with immunized mice (**Figure 13A**). These histopathological findings were consistent with the reduced serum activity of the cardiac injury marker CK-MB observed in vaccinated animals (**Figure 11**).

To directly assess protection during the chronic phase of infection, parasite burden in skeletal and cardiac muscle was quantified by real-time PCR at 120 days post-infection. All immunized groups exhibited marked reductions in parasite load in both target tissues compared with the control group, indicating sustained protection beyond the acute phase (**Figure 13B**). In skeletal muscle, parasite burden was reduced 3- to 5-fold, with statistically significant decreases observed in the SCruSEG and rCruSEG+CpG groups. In cardiac tissue, reductions ranged from 2- to 4-fold relative to controls, with significant differences detected in the rCruSEG+CpG and prime–boost groups.

Taken together, these results demonstrate that immunization with CruSEG, using the different vaccination schemes evaluated, significantly improved parasite control during both the acute and chronic phases of *Trypanosoma cruzi* infection. This protective effect was reflected by reduced parasite burdens in target tissues and was accompanied by a marked decrease in tissue damage, as evidenced by lower serum levels of tissue injury markers and reduced histopathological alterations.

## Discussion

Building on previous work demonstrating the immunoprotective capacity of the heterologous chimeric antigen rCruSEG as a subunit vaccine (24), the present study explored an alternative vaccination strategy based on DNA delivery using an attenuated *Salmonella* vector, with the objective of enhancing cellular immune responses. DNA vaccination platforms are known to preferentially promote Th1-biased immunity and strong cellular responses, which are particularly relevant for the control of intracellular pathogens such as *T. cruzi* (34). In this context, attenuated *Salmonella* strains have been extensively validated as antigen and DNA delivery systems due to their intrinsic adjuvant properties, genetic stability, and capacity to activate innate immune pathways that shape adaptive immunity (35). Consistent with previous studies from our laboratory using different *T. cruzi* antigens (12–16), the SCruSEG platform proved effective in inducing immune responses associated with parasite control.

Analysis of humoral immune responses revealed that immunization with recombinant CruSEG formulated with CpG induced high titers of Nt-Cz–specific IgG antibodies, both in homologous regimens and in the heterologous prime–boost strategy. In contrast, oral immunization with SCruSEG resulted in low serum antibody titers as measured by indirect ELISA. This observation is consistent with previous reports indicating that oral vaccination with attenuated *Salmonella* frequently induces modest systemic antibody titers despite robust cellular immunity (9, 14, 15, 36, 37). In the prime–boost scheme, the protein boost formulated with CpG likely accounts for the enhanced antibody titers relative to homologous SCruSEG immunization.

Despite quantitative differences in antibody titers, all vaccination protocols induced a predominance of IgG2a over IgG1 antibodies, indicative of a Th1-skewed humoral response. This profile is considered particularly favorable for protection against intracellular pathogens such as *T. cruzi*. Similar Th1 polarization has been reported for heterologous DNA/protein prime–boost strategies in other infectious models, where the sequence of vaccine platforms critically influences the quality of the immune response (38–40).

Importantly, sera from all vaccinated groups efficiently inhibited parasite invasion of non-phagocytic cells *in vitro*, underscoring the functional quality of the antibody responses elicited. This finding is consistent with the established role of cruzipain in host cell invasion and its localization at the parasite surface (41). Previous studies from our group have demonstrated that antibodies directed against the N-terminal domain of cruzipain possess both neutralizing and complement-mediated lytic activity (23, 30), supporting their contribution to parasite control *in vivo*.

Notably, sera from mice immunized with SCruSEG displayed strong neutralizing activity despite low antibody titers detected by ELISA. The presence of antigen-specific antibodies in this group was confirmed using a sensitive SPR-based capture assay, revealing specific interactions between immune sera and rCruSEG. This discrepancy likely reflects differences in assay sensitivity and epitope presentation, as well as the possibility that DNA immunization induces antibodies present at lower abundance but with higher affinity. Similar observations have been reported for other *T. cruzi* antigens delivered using attenuated *Salmonella* vectors (12, 14, 15), reinforcing the notion that antibody functionality rather than titer alone represents a relevant correlate of protection.

The cellular immune responses elicited by the different vaccination protocols further highlighted the advantages of the DNA delivery strategy. Both *in vivo* and *in vitro* assays demonstrated that SCruSEG immunization induced stronger antigen-specific cellular responses than recombinant protein vaccination alone, as evidenced by enhanced delayed-type hypersensitivity reactions and increased antigen-specific splenocyte proliferation. These responses were accompanied by a Th1-skewed cytokine profile characterized by increased secretion of IFN-γ and IL-12 following antigenic restimulation.

IL-12 plays a central role in resistance to *T. cruzi* infection by promoting Th1 differentiation and enhancing macrophage microbicidal activity through IFN-γ– and TNF-α–dependent mechanisms (42). IFN-γ itself is a key effector cytokine in Chagas disease, contributing to macrophage activation, potentiation of CD8^+^ T cell responses, and immunoglobulin class switching toward protective IgG2a isotypes. In parallel, all vaccination protocols induced IL-10 secretion upon antigenic stimulation, with higher levels observed in the prime–boost group. IL-10 exerts a dual role during *T. cruzi* infection, contributing to the control of immunopathology while potentially limiting excessive inflammatory responses (43, 44), suggesting that the balanced cytokine profiles observed may favor parasite control while mitigating tissue damage.

CruSEG-based vaccination induced a robust and highly functional CD4^+^ T cell response dominated by polyfunctional cells producing IFN-γ, TNF-α, and IL-2. Polyfunctional CD4^+^ T cells have been consistently associated with effective immune control of chronic infections caused by intracellular pathogens (31, 45). In the context of Chagas disease, polyfunctional CD4^+^ T cells correlate with early or controlled infection, whereas chronic disease progression is characterized by functional exhaustion and predominance of monofunctional IFN-γ–producing CD4^+^ T cells (46, 47). Similar polyfunctional CD4^+^ T cell responses have been reported following vaccination with other *T. cruzi* antigens, including Tc80 and Traspain (15, 16), underscoring their role as critical correlates of vaccine-mediated protection.

Beyond the frequency of responding cells, qualitative differences in cytokine production were observed among vaccination strategies. Although the CruSEG+CpG protocol induced robust CD4^+^ T cell activation, lower IFN-γ secretion in culture supernatants reflected reduced per-cell cytokine production, as shown by MFI and iMFI analyses. These findings emphasize the importance of integrating both quantitative and qualitative parameters when evaluating vaccine-induced T cell responses.

Regarding CD8^+^ T cell responses, SCruSEG immunization induced the highest frequencies of IFN-γ–producing CD8^+^ T cells, consistent with the ability of DNA vaccines to efficiently prime this compartment. Although these responses were predominantly monofunctional, IFN-γ–producing CD8^+^ T cells have been shown to contribute significantly to parasite control in experimental *T. cruzi* infection (48). Moreover, CD8^+^ T cell effector functions in the absence of adequate CD4^+^ T cell help are insufficient for effective parasite control (49), underscoring the relevance of the coordinated CD4^+^/CD8^+^ responses observed in this study.

The protective efficacy of the vaccination protocols was evaluated using two complementary challenge models. Acute infection with the highly virulent RA strain revealed that vaccination with four doses of SCruSEG or rCruSEG+CpG resulted in the most pronounced reductions in circulating parasite levels and improved clinical parameters such as body weight maintenance. Protection was further assessed using the K98 clone, a less virulent and myotropic strain that allows evaluation of both acute parasitemia and chronic infection. All vaccination protocols significantly reduced parasitemia during the acute phase, and this protection extended into the chronic phase, as evidenced by reduced parasite burdens in skeletal and cardiac muscle.

Reduced tissue parasitism correlated with lower serum levels of muscle damage markers and diminished histopathological alterations, in agreement with previous studies linking parasite persistence with inflammatory tissue damage during chronic Chagas disease (50, 51). Greater involvement of skeletal muscle compared with cardiac tissue was observed, consistent with experimental evidence indicating that chronic myositis represents a relevant but often underappreciated component of Chagas pathology (52).

From a translational perspective, although the heterologous prime–boost strategy generated a broader immune profile, the levels of protection achieved were comparable to those obtained with homologous vaccination protocols using either recombinant protein or DNA delivery alone. This observation indicates that heterologous prime–boost regimens, while valuable for dissecting immune mechanisms and optimizing response quality, may not represent the most practical option for large-scale implementation. In this context, the SCruSEG and rCruSEG+CpG protocols emerge as viable and potentially more cost-effective alternatives, each relying on distinct but effective immune mechanisms.

In summary, this study demonstrates that CruSEG-based vaccination strategies combining antigen specificity with immune modulation (53) are capable of inducing coordinated humoral and cellular immune responses that translate into effective control of *T. cruzi* infection (54). Although sterilizing immunity was not achieved, the sustained reduction in parasite burden and attenuation of tissue damage represent biologically and clinically meaningful outcomes. A central conceptual advance of this work is the validation of the CruSEG chimera as a platform-independent immunogen. The successful transition from a recombinant protein to a *Salmonella*-mediated DNA delivery system demonstrates that the genetically detoxified superantigen SEGN24A functions as a robust and versatile immune modulator, irrespective of whether it is administered as a chimeric protein or encoded within a DNA vector.

## Concluding remarks

Different vaccination strategies have been explored for chronic infectious diseases, each presenting specific advantages and limitations depending on their intended application. In the past five years, advances in vaccine delivery have driven science and public health into a new era of vaccine design. Nucleic acid vaccines delivered by biological systems, nanoparticles as carriers, or even acting as immunogens themselves have established a new generation of vaccines. In this context, engineered chimeric antigens, which combine distinct immunogenic and protective domains from different proteins of the same or different pathogens, may constitute the basis of polyvalent vaccines. Over the last decade, the development of adjuvants functioning as immune modulators has become a powerful tool to induce the type of immune response that is theoretically protective. Superantigens, potent toxins, have also been modified with the aim of treating malignant processes. In this manuscript, we designed a chimeric immunogen that fuses the protective domain of cruzipain, a *T. cruzi* protease, with an attenuated superantigen capable of eliciting a type 1 immune response, which has been shown to be protective during the acute and early chronic phases of *T. cruzi* infection in a mouse model. The promising protective and immunological outcomes observed here support further optimization of this chimeric vaccine platform. Future research directions include refinement of antigen design, comprehensive toxicity studies, exploration of heterologous prime–boost strategies and delivery systems, and evaluation of different routes of administration to enhance protective efficacy. In addition, extending follow-up periods and incorporating complementary animal models will be essential to better define long-term protection and translational potential. Collectively, these results will contribute to the rational development of optimized vaccination strategies for chronic infectious diseases.

## Data Availability

The raw data supporting the conclusions of this article will be made available by the authors, without undue reservation.

## Supplementary material

Supplementary Figure 1 Surface plasmon resonance (SPR) sensorgrams showing specific interactions between CruSEG and immune sera. CruSEG (30 mg/mL) was captured on a CM5 chip via immobilized anti-SEG IgG (6600 RU), followed by injection of serial dilutions of immune sera from the SCruSEG (A) and prime boost (B) groups; serum dilutions are indicated on the right.
