# Supplementary figures and images for: Protective immunity against Chagas disease induced by a superantigen-based chimeric DNA vaccine delivered by attenuated Salmonella
Source: Front Immunol. 2026 Mar 12;17:1788924. doi: 10.3389/fimmu.2026.1788924 (PMC13018133; doi:10.3389/fimmu.2026.1788924)

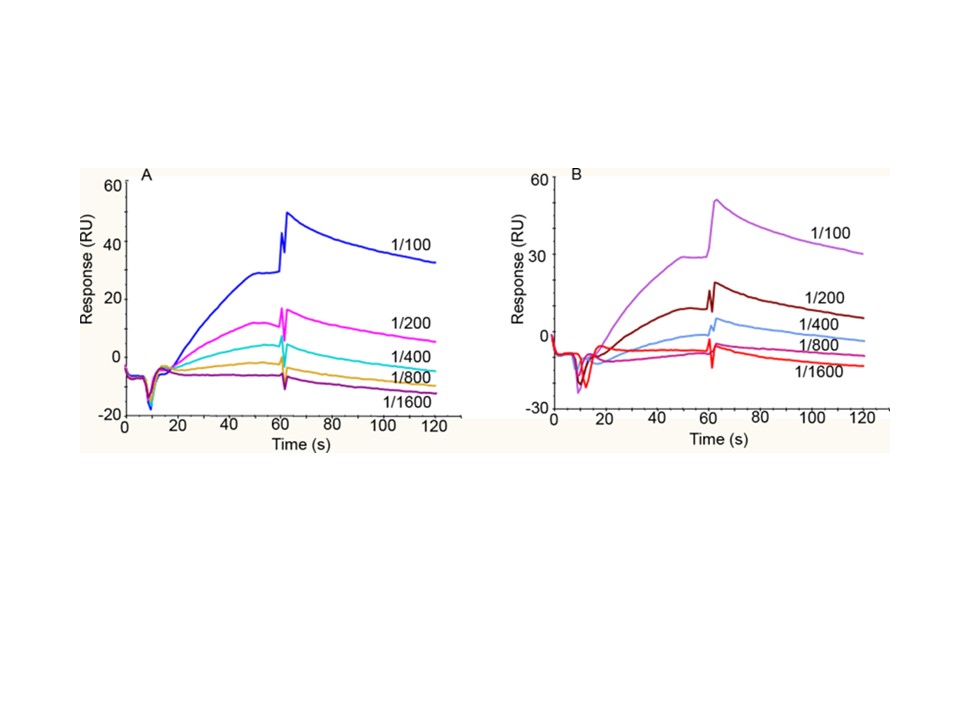

Supplement: Supplementary Figure 1 — Surface plasmon resonance (SPR) sensorgrams showing specific interactions between CruSEG and immune sera. CruSEG (30 mg/mL) was captured on a CM5 chip via immobilized anti-SEG IgG (6600 RU), followed by injection of serial dilutions of immune sera from the SCruSEG (A) and prime boost (B) groups; serum dilutions are indicated on the right. [file Image1.jpeg]
